# Trends and Characteristics of Emergency Medical Services in Italy: A 5-Years Population-Based Registry Analysis

**DOI:** 10.3390/healthcare8040551

**Published:** 2020-12-11

**Authors:** Sara Campagna, Alessio Conti, Valerio Dimonte, Marco Dalmasso, Michele Starnini, Maria Michela Gianino, Alberto Borraccino

**Affiliations:** 1 Department of Public Health and Paediatrics, University of Torino, 10126 Torino, Italy; sara.campagna@unito.it (S.C.); alessio.conti@unito.it (A.C.); valerio.dimonte@unito.it (V.D.); alberto.borraccino@unito.it (A.B.); 2 Epidemiology Unit, Local Health Unit TO3, Piedmont Region, 10195 Grugliasco, Italy; marco.dalmasso@epi.piemonte.it; 3 Institute of Scientific Interchange (ISI) Foundation, 10126 Torino, Italy; michele.starnini@isi.it

**Keywords:** emergency medical services, information systems, prehospital intervention, population surveillance

## Abstract

*Background:* Emergency Medical Services (EMS) plays a fundamental role in providing good quality healthcare services to citizens, as they are the first responders in distressing situations. Few studies have used available EMS data to investigate EMS call characteristics and subsequent responses. *Methods:* Data were extracted from the emergency registry for the period 2013–2017. This included call and rescue vehicle dispatch information. All relationships in analyses and differences in events proportion between 2013 and 2017 were tested against the Pearson’s Chi-Square with a 99% level of confidence. *Results*: Among the 2,120,838 emergency calls, operators dispatched at least one rescue vehicle for 1,494,855. There was an estimated overall incidence of 96 emergency calls and 75 rescue vehicles dispatched per 1000 inhabitants per year. Most calls were made by private citizens, during the daytime, and were made from home (63.8%); 31% of rescue vehicle dispatches were advanced emergency medical vehicles. The highest number of rescue vehicle dispatches ended at the emergency department (74.7%). *Conclusions*: Our data showed that, with some exception due to environmental differences, the highest proportion of incoming emergency calls is not acute or urgent and could be more effectively managed in other settings than in an Emergency Departments (ED). Better management of dispatch can reduce crowding and save hospital emergency departments time, personnel, and health system costs.

## 1. Introduction

Emergency Medical Services (EMS) systems primary function is to deliver emergency medical care in all emergencies, including disasters; it is a system of coordinated response, involving multiple people and agencies. An efficient EMS system can help reduce injuries and mortality and is essential to ensure a prompt emergency response to urgent situations of illness or trauma.

Out-of-hospital emergency medical services is a subsystem of EMS and refers to the delivery of medical care at the site of the adverse medical event [1].

Through a dispatch centre, out-of-hospital EMS answers emergency calls provide medical advice to the caller and, if needed, dispatches a mobile medical care unit. 

As a first response provider, out-of-hospital EMS must contribute to the reduction of unnecessary pressures on Emergency Departments (ED), through direct transportation to a more appropriate destination, by providing necessary care on-scene, or by providing advice over the telephone. Offering such alternatives has the potential to reduce pressures on the system but can also benefit patients by ensuring they receive ‘the right treatment, at the right time, by the right person’ [2].

Even though EMS systems have many similarities there is no common European or US standard agreed upon; in general, the EMS status of a country depends on its peculiar geographic, political, cultural, linguistic, historical and medical setting [1].

Given the heterogeneity of EMS systems, it is important to collect data to understand environmental management at a local level, and to disseminate data to health professionals and policymakers. This point is particularly important in times of the Covid-19 pandemic crisis, where information flow between countries is vital for the cross-border interoperability of EMSs and effective coordinated response to crises.

The most recent information available on the Italian EMS in Italy dates back to a 2005 survey, which was published by the Ministry of Health in 2007 and highlighted the wide regional differences in several aspects of EMS response [3]. Few recent studies have attempted to study the EMS response characteristics within specific health problems [4,5,6,7,8], in relation to time of response [9,10] or about the accuracy of the data bases available [11]. 

In 2010 the Ministry of Health, in Italy, renewed its model of assistance [12]. The model points towards the enhancement of health care activities in the local area and the development of a network of specialised structures designated to the management of complex pathologies. This new model is further aimed to improve the regional EMS network. The territorial EMS network is the second level Public-Safety Answering Point (PASP) provided with a main operative centre, in charge of answering incoming calls, assessing the severity and activating the vehicle dispatch, and several local operation stations where staff and different types of rescue vehicles are allocated. Although EMS systems are organised according to national guidelines, they have regional autonomy, resulting in several different EMS response models [3,13]. 

The recent pandemic has forced policymakers to reconsider EMS systems deeply and quickly, to better manage an increased number of calls, to avoid a large flow of people into EDs and, at the same time, to enhance population care on site by improving primary care [14,15]. 

The services have been revised and soon reorganised to respond to the crisis without having the opportunity to reflect on the previous arrangement. Reconsidering the system’s strengths and limitations at the local level before the pandemic will help decision-makers to make more appropriate and long-lasting structural changes to better meet the population needs. To the authors’ knowledge, no studies in Italy have used available EMS data since its renewal to contribute to the improvement of a timely cost-effective EMS response. 

Given these premises, the purpose of this study is to analyse trends and characteristics of incoming emergency calls and corresponding EMS responses in one of the largest regions of Italy, to identify weaknesses of the pre-existing system in responding to emergency needs of the population.

## 2. Materials and Methods 

### 2.1. Study Setting

The Piedmont Region is the second largest of Italy’s 20 regions, measuring 25,300 km^2^, and it ranks seventh in number of inhabitants (nearly 4.3 million in 1182 municipalities). The EMS in Piedmont consists of one regional operation centre and four operative stations, all of which are coordinated by a medical doctor and managed by nurses. There are three types of rescue vehicles available: advanced emergency medical vehicles (EMVs), basic EMVs, and one rescue helicopter for each centre. *Advanced EMVs* carry one of two rescue teams: type (1) doctors and nurses with advanced life-saving skills, as well as specially trained volunteer rescuers or type (2) nurses with advanced life-saving skills as well as specially trained volunteer rescuers. *Basic EMVs* carry a rescue team of specially trained volunteer rescuers with basic life support skills. Rescue *helicopter* teams consist of an anaesthesiologist, a nurse, and a mountain rescue technician. 

Incoming emergency calls are received by trained nurse operators in the 2nd level PSAP and assessed by means of a dynamic decision-making tool, the Medical Priority Dispatch System (MPDS). This systematic, digital program is structured to guide nurses in helping the caller to provide the required information, in determining the appropriate intervention (dispatch of a specific rescue vehicle or provide phone advice) and, in case of need, in instructing the rescue team to better respond to the identified health demands. It also assists nurses in assigning the correct EMS criticality code (red: need of an immediate response, life-threatening situation with a chance of survival; yellow: rapid response, presence of a possible life-threatening condition not in an immediate danger of death; green: the situation is not an emergency, presence of injuries, acute but stable conditions; and white: the situation is not an emergency, minor injuries not a life-threatening situation, medical care not often required) and in determining which rescue vehicle should be dispatched. A second nurse then locates and dispatches the requested rescue vehicle, based on the EMS criticality code and vehicle availability. 

### 2.2. The Emergency-Urgency Registry

Since 2012, selected regional MPDS data have been entered into the EMUR (EMergency-URgency) registry, which is the anonymised, official Ministerial Health Regional Database of all EMS interventions. The registry contains information on 70 different variables that cover incoming emergency calls, the type of intervention employed (phone advice, dispatch of a rescue vehicle, etc.), the rescue vehicle activation process, intervention outcomes, and patient information. Of these 70 variables, 51% (36 variables) are compulsory. Data are checked at the regional level for logical consistency and completeness before they are officially released for administrative or epidemiological purposes. The EMUR registry can be used, without further authorisation, for any epidemiological study. Moreover, the Regional Public Health Observatory, as a member of the Public Health Department (Local Health Board TO3), together with the University of Torino, have access to the EMUR registry; therefore, ethics committee approval was not required.

Unfortunately, some weaknesses embedded in the current structure of the EMUR registry impede that an individual can be easily tracked throughout the whole process particularly when two or more rescue vehicles are dispatched for the same emergency call and/or when two or more patients are moved using the same rescue vehicle. For these reasons, to avoid any inconsistencies, the present analysis focused specifically on the EMS process, without detailing information on patient characteristics.

### 2.3. Data Selection and Analysis

The analysis assessed frequencies and characteristics of incoming emergency calls that ended with the dispatch of at least one rescue vehicle during a 5-year study period (2013–2017). Data for this period were extracted from the EMUR registry, including call information: caller, call time, call location; and rescue vehicle dispatch information: number of people involved, health problem/injury reported by the caller (classified by the operator according to International Classification of Disease (ICD) standard codes), type of rescue vehicle dispatched, number of rescue vehicles dispatched, and site of rescue completion. Data on the 2013–2017 population of the Piedmont Region were extracted from the public regional census database freely available on the net. All relationships in analyses and differences in events proportions between 2013 and 2017 were tested against the Pearson’s Chi-Square with a 99% level of confidence. The likelihood of completing a rescue into a hospital ED by type of rescue vehicles in relation to the Emergency Medical Services (EMS) criticality codes assigned was evaluated by a set of logistic regressions. Results were presented as odds ratios (ORs) and corresponding 95% confidence intervals (CIs) adjusted for all other variables. Population estimates were reported as a rate per 1000 inhabitants. All analyses were performed in Stata version release 14 (Stata Corp, College Station, TX, USA).

## 3. Results

**Figure 1:**
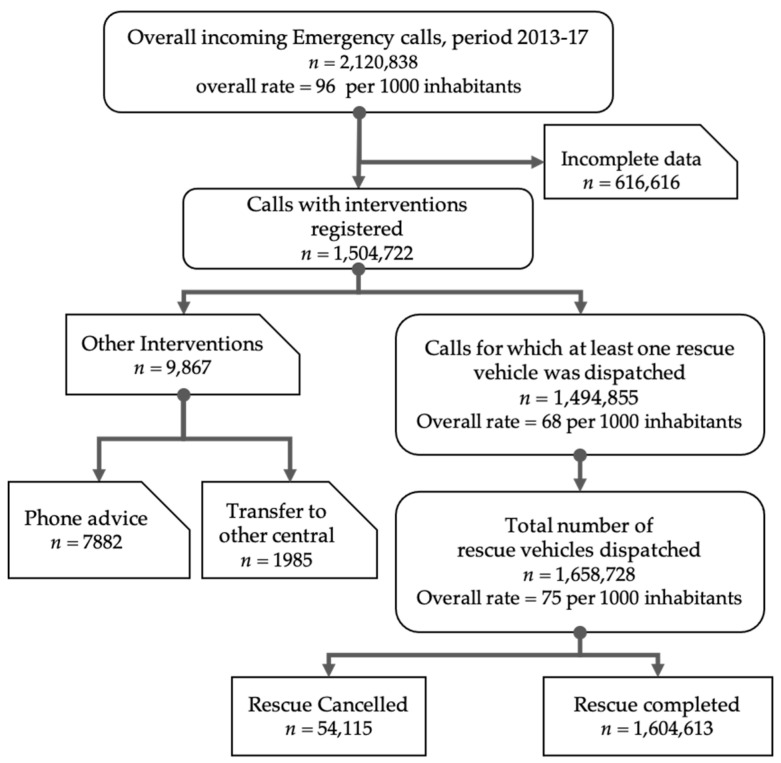
Study flow of incoming emergency calls and corresponding Emergency Medical Services (EMS) responses.

A total of 2,120,838 incoming emergency calls were reported in the EMUR registry for the Piedmont Region during the study period (Figure 1). Among these, 1,504,722 (71%) calls required some level of intervention of which 99.4% (*n* = 1,494,855) required the dispatch of at least one rescue vehicle, generating the dispatch of 1,658,728 rescue vehicles in total. In the remaining 9867 (0.6%) calls, the caller was given simple phone advice or that call was labelled “transferred to another operative station” for regional competency. 

### 3.1. Trends in EMS Use 

Between 2013 and 2017 emergency calls increased by 34.2% (from 240,878 to 323,155) and the population reduced by 1.4% over the 5-year period. The estimated overall incidence of emergency calls was 96 per 1000 inhabitants per year, which nearly doubled (+45.5%) from 55 calls in 2013 to 102 calls per 1000 inhabitants in 2017. There was an average of 75 rescue vehicles dispatched per 1000 inhabitants per year, with an increase of about 40%, from 59 vehicles dispatched per 1000 inhabitants in 2013 to 83 in 2017.

Overall, nearly 90% of the 1,494,855 calls that required the dispatch of at least one rescue vehicle were made by private citizens (Table 1 and Table S1 in Supplementary Materials), two-thirds occurred between 7 a.m. and 6:59 p.m. and 71.6% in working days. The 63.8% were made from a home, although a 2% decrease was observed during the study period. This slight decrease in calls from a home was observed in parallel to a significant slight annual increase in calls from other locations, i.e., nursing homes, private hospitals, and residential care facilities (from 15.3% in 2013 to 16.7% in 2017). Calls from streets or highways or any other location remained stable at around 13%.

### 3.2. Trends in Dispatch 

When looking at the 1,658,728 rescue vehicles dispatched (Table 2 and Table S2 in Supplementary Materials), around 30% were advanced EMVs, with a slight significant decrease in advanced EMV type 1 vehicles; the remaining were basic EMVs (around 68% of all dispatches), and rescue helicopters significantly increased from 0.4% (95%CI 0.39%–0.44%) in 2013 to 0.7% (95% CI 0.67%–0.72%) in 2017 (see also Supplementary Materials, Table S3).

The most common rescue completion site was a hospital ED with a slight significant decrease between 2013 (76.6%) and 2017 (73.2%). Nearly a quarter of all dispatched rescue vehicles reported no completion site for one of the following reasons: vehicle failure, patient not found or already transported, site unattainable, or rescue cancellation by the operation centre.

Of all dispatches, the range of health problem/injury reported by the caller over the study period remained roughly stable, and traumatic events, cardiocirculatory, neurological, or respiratory problems accounted for the about the 60% of all complaints; “unclear problems” represented the 26% of all dispatches without variations between 2013 and 2017. 

**Figure 2:**
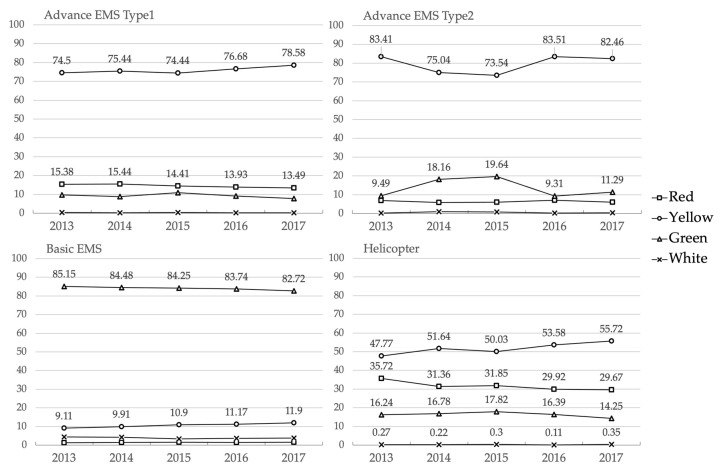
Proportional frequencies (%) of rescue completion (RC) at a hospital emergency department (ED) by Emergency Medical Services (EMS) criticality codes (Red, Yellow, Green, White) for type of rescue vehicle dispatched: Advanced EMS vehicle type1) doctors and nurses with advanced life-saving capabilities, as well as specially trained volunteer rescuers or type2) nurses with advanced life-saving capabilities as well as specially trained volunteer rescuers. Basic EMS vehicle carry a rescue team of specially trained volunteer rescuers with basic life support capabilities. Rescue helicopter teams consist of an anaesthesiologist, a nurse, and a mountain rescue technician. RC—rescue completion; HED—hospital emergency department.

Of all the rescues that ended in a hospital ED (Figure 2), Basic EMVs were dispatched more frequently for situations that were given non-urgent criticality codes (from 85.1 to 82.7% among the whole period). Advanced EMVs with a crew of nurses and doctors (type 1), and those with a nurse on board (type 2) were dispatched more frequently for situations that were given urgent criticality codes and around the 10.6% and an average of 15.2%, respectively, for non-urgent cases. 

A rescue helicopter was dispatched in more than the 80% of urgent cases.

Regression analysis, reported in Table 3, showed an increased likelihood of rescue completions into a hospital ED in all type of criticality codes for all vehicles with nurse or only volunteers’ crew. Basic EMV had a higher likelihood of ending into a hospital ED for non-urgent codes when compared to advanced vehicles type (OR 3.21, 95%CI 3.1–3.3). Helicopter 

## 4. Discussion

To the authors’ knowledge, this is the first study that, using ministerial data, reported attempted characteristics of EMS responses and trends in one of the 20 Italian regions, over a period of 5 years. Previously published studies have been limited to 1-year or to single-city analyses [3,7].

Based on the available information, the EMUR registry provided a robust representation of EMS use and, despite its deficiencies, it showed to be a valid and suitable tool to allow a comprehensive characterisation of the emergency response system. 

Our results showed an increase in the EMS use over a 5-years period. This result is supported by previous studies highlighting that the demand of EMS has substantially increased over time in developed countries like Spain [16], UK [17], Switzerland [18], Japan [19], USA [20] and Australia [21]. These studies have also linked the EMS demand increase to the concurrent population growth. Unfortunately, over the studied period the overall Piedmont population reduced of about 1.4% suggesting that the observed increase in EMS use had to be attributed to others drivers, as the ageing of the population [22,23] and the increased frailty of the community-dwelling of the older people [24]. Hypothesis which, in this study, can be further supported by the increased trend of dispatches coming from long term care facilities and the higher prevalence of medical conditions and of non-urgent codes. 

Coherently with other studies, medical problems as cardiocirculatory, neurological, and respiratory, accounted for the largest proportion of EMS intervention requests [25]. Interestingly, given the robustness of our data, although each incoming emergency call is preliminary handled by a highly qualified nurse further supported by a computerised MPSD, “unclear problems” accounted for nearly a quarter of the total rescue vehicle dispatched, with little variations by year. Studies on the occurrence of such calls in the EMS are sparse and reported some variations ranging from 11% in Norway [26], to 18% in Denmark [27], to nearly 20% (2 million unknown or non-reported complaints over more than 10 million calls) in the USA National Emergency Medical Services Information System (NEMSIS) population [28]. The high occurrence of unclear problems in Italy could be also due to the EMUR registry internal structure, as it uses ICD9 codes to categorize requests, which are meant to diagnose and classify pathologies and may not be accurate enough to identify the callers’ complaints in emergency situation. In other contexts, the emergency situation is classified by using a chief complaint classification codes [29,30]. This approach that is based on specific caller’s complaints is likely to be more accurate, as it defines the situation or visible problem as judged on site by a non-professional, resulting in a lower number of unidentified/unclear conditions. Regardless of the reasons, when the cause of a call is reported as unclear, the risk stratification may be less sensitive, leading to the assignment of a higher or lower EMS criticality code than the one needed [29]. This can result in an unnecessary response or in an unmatched response, with more advanced rescue vehicles being sent in basic emergencies, and vice-versa. As a consequence, resultant unneeded EMS transports could affect hospital workloads and reduce the number of cases that could be effectively managed on-site through the dispatch of a more appropriate vehicle. Indeed, Møller (2017) found a higher mortality rate (incidence rate ratio 1.26; 95% confidence interval 1.18-1.36) in patients whose calls were registered as unclear complaints, denoting the need to improve the classification system in order to provide a better, more effective response [27].

Our results showed that the greatest number of calls was from private citizens, the main call location was a private home, occurring in daytime and receiving a low criticality code. Such findings may highlight the existence of problems that could be more effectively managed in primary care, the recurrence of which may also reveal the existence of barriers to primary care services access. Reviews studies on this topic had showed that the lack of a primary care system able to manage primary care sensitive problems were related to an increased EMS services [23,31]. 

Due to national indications, the Piedmont health system underwent a heavy spending review from 2010 to 2017 [32] which has hugely affected on the organization of primary care services. The regulatory action has impacted on the number of annual recruitments that, coupled with the increase in the workforce shortage, has dramatically and reduced GPs and nurses’ availability and access. 

Of all the dispatches in the Piedmont Region, more than two third of all dispatches employed Basic EMVs. It is difficult to agree on whether this organisational approach can be considered appropriate or not, without any available standards and since each regional authority is allowed to manage its EMS autonomously. In the Marche Region, for example, advanced EMVs were employed in nearly 70% of emergency calls, while other regions showed overlapping responses to those observed in our study [13]. 

The EMS system is expected to provide the right answer to the patients’ health conditions, transporting only those truly requiring a hospital ED intervention. As the transport is likely to be appropriate in patients receiving an urgent critical code, green codes occurrence should be further analysed. Given that Basic EMVs crew is only allowed to transport patients, calls receiving a non-urgent code can only be dispatched into a hospital ED, independently of the health need. As green codes accounted for nearly 800,000 transports over the 5 years period, the 86% of them had probably contributed to the ED overcrowding. With some exception, a huge proportion of such incoming calls may more effectively managed in a primary care setting than in an emergency department, saving hospital ED time and reducing health system costs [25]. Moreover out-of-hospital EMS reorganization, as for example by providing necessary care on-scene, or by providing advice over the telephone, as occurred in the recent COVID-19 crisis, could contribute to the reduction of unnecessary pressures on ED personnel [33]. The need to further foster primary care is, indeed, supported by the evidence that under the COVID scenario the Italian government (D.L. 14 del 9/3/2020.) intervened in reorganising the EMS system response. To avoid massive ED accesses new pathways of care and professional teams, such as USCA (Special Unit for Continuity of Care), were activated to better respond the health demand and to avoid unnecessary or spontaneous hospital accesses [34].

### Study Limits

The main study limitations are those linked to the database that was used and are common to all administrative database studies. Due to the EMUR weaknesses, at present, it is not possible to properly link the EMS registry with hospital health data, which is a critical limitation. Moreover, the EMUR registry does not currently include emergency department information, nor data on patient characteristics, such as age, civil status (alone or cohabiting), symptom information, or medications administered. At the same time, the current EMUR registry database represents the largest, affordable and representative collection of data characterising EMS care in Italy. The Italian health care system is characterized by the universalism of care that is guaranteed to the whole population and therefore the results cannot be generalized to other health care systems.

## 5. Conclusions

In conclusion, the EMUR registry is useful in characterising EMS systems, level of intervention, and prehospital patient needs. Piedmont regional EMS dispatches mainly Basic EMVs that were employed in low criticality codes or as a support; consequently, these vehicles can only drive the patient to the hospital. Our data showed that, with some exception due to environmental differences, the highest proportion of incoming emergency calls is not acute or urgent and could be more effectively managed in other settings than in an ED. Better management of dispatch can reduce crowding and save hospital emergency departments time, personnel, and health system costs [35,36]. 

This data highlights the importance of promoting policies to increase the availability of ambulances with staff who can manage problems on site, reducing the admissions of less appropriate EDs. 

Moreover, specific procedures to equip EM operating centres in providing updated information on the location and the availability of EMVs fleet, as well as, in receiving indications on the availability of beds in the nearest local hospitals is becoming a priority specifically in densely population regions. Finally, the slight increase in calls coming from nursing homes highlights the need to define priority paths between the local care facilities and the hospitals to reduce ED waiting times.

## Figures and Tables

**Table 1:** Characteristics of Emergency Medical Services (EMS) calls for which at least one rescue vehicle was dispatched, by year (2013–2017).

EMS Calls	2013	2014	2015	2016	2017	Overall	*p*
Characteristics	*N* (%)	*N* (%)	*N* (%)	*N* (%)	*N* (%)	*N* (%)	Value
Private citizen	210,433 (87.4)	228,106 (87.0)	283,699 (86.8)	297,849 (87.2)	286,627 (88.7)	1,306,714 (87.4)	<0.001
Nursing home	8181 (3.4)	8941 (3.4)	11,364 (3.5)	10,401 (3.0)	10,026 (3.1)	48,913 (3.3)	
Authority	6973 (2.9)	8127 (3.1)	10,539 (3.2)	12,086 (3.5)	8459 (2.6)	46,184 (3.1)	
GP/Paediatrician	6039 (2.5)	6823 (2.6)	7991 (2.4)	7614 (2.2)	5594 (1.7)	34,061 (2.3)	
EMS Physician	5978 (2.5)	7030 (2.7)	7690 (2.3)	6651 (1.9)	4680 (1.4)	32,029 (2.1)	
Private hospital	516 (0.2)	504 (0.2)	581 (0.2)	484 (0.1)	515 (0.2)	2600 (0.2)	
Other ^†^	2758 (1.1)	2679 (1.0)	5127 (1.6)	6536 (1.9)	7254 (2.2)	24,354 (1.6)	
**Call Time**							
7a.m.–6:59p.m.	155,715 (64.6)	170,219 (64.9)	213,938 (65.4)	222,640 (65.2)	211,154 (65.3)	973,666 (65.1)	<0.001
7p.m.–6:59a.m.	85,163 (35.4)	91,991 (35.1)	113,053 (34.6)	118,981 (34.8)	112,001 (34.7)	521,189 (34.9)	
**Day of the Call**							
Working days	172,146 (71.5)	187,564 (71.5)	234,607 (71.8)	245,107 (71.8)	231,195 (71.5)	1,070,619 (71.6)	<0.001
Weekend	68,732 (28.5)	74,646 (28.5)	92,384 (28.3)	96,514 (28.3)	91,960 (28.5)	424,236 (28.4)	
**Call Location**							
Home	156,058 (64.8)	170,211 (64.9)	208,232 (63.6)	215,458 (63.1)	203 105 (62.8)	953,064 (63.8)	<0.001
Street or highway	30,505 (12.7)	33,399 (12.7)	41,996 (12.8)	45,540 (13.3)	42,258 (13.1)	193,698 (13.0)	
Public building	8421 (3.5)	9114 (3.5)	11,933 (3.6)	12,667 (3.7)	11,749 (3.6)	53,884 (3.6)	
Industrial place	4529 (1.9)	5046 (1.9)	6 706 (2.0)	6686 (2.0)	6197 (1.9)	29,164 (1.9)	
Sport building	2386 (1.0)	2578 (1.0)	3 100 (1.0)	3048 (0.9)	2950 (0.9)	14,062 (0.9)	
School	2109 (0.8)	2568 (1.0)	2 807 (1.0)	3026 (0.9)	3009 (1.0)	13,519 (0.9)	
Other locations *	36,870 (15.3)	39,294 (15.0)	52,217 (16.0)	55,196 (16.1)	53,887 (16.7)	237,464 (15.9)	
Total EMS Calls	240,878	262,210	326,991	341,621	323,155	1,494,855	

^†^ Other Callers comprise all calls originally labelled as “none of the above”; * Other Call locations comprise nursing homes, private hospitals, and residential care facilities; GP: general practitioner.

**Table 2:** Characteristics of Emergency Medical Services (EMS) rescue vehicle dispatches by year (2013–2017) among 1,658,728 total rescue vehicles dispatched.

EMS Dispatches	2013	2014	2015	2016	2017	Overall	*p*
Characteristics	*N* (%)	*N* (%)	*N* (%)	*N* (%)	*N* (%)	*N* (%)	Value
Number of People Involved							
1	223,558 (85.5)	241,632 (83.8)	297,661 (81.7)	306,942 (80.3)	289,034 (79.8)	1,358,827 (81.9)	<0.001
2	36,740 (14.0)	45,018 (15.6)	61,527 (16.9)	67479 (17.6)	65,505 (18.1)	276,269 (16.7)	
>2	1221 (0.5)	1638 (0.6)	5249 (1.4)	7961 (2.1)	7563 (2.1)	23,632 (1.4)	
Health Problem/Injury Reported by the Caller							
Traumatic	52,858 (20.2)	57,993 (20.1)	82,319 (22.6)	87,333 (22.8)	82,478 (22.8)	362,981 (21.9)	<0.001
Cardiocirculatory	40,485 (15.5)	43,420 (15.1)	53,185 (15.6)	55,399 (14.5)	58,406 (16.1)	250,895 (15.1)	
Neurological	32,083 (12.3)	36,121 (12.5)	43,742 (12.0)	45,622 (11.9)	40,798 (11.3)	198,366 (12.0)	
Respiratory	30,513 (11.7)	33,372 (11.6)	41,803 (11.5)	42,060 (11.0)	40,836 (11.3)	188,584 (11.4)	
Psychiatric	8370 (3.2)	9508 (3.3)	11214 (3.1)	12,066 (3.2)	11,196 (3.1)	52,354 (3.2)	
Toxicological	4560 (1.7)	4704 (1.6)	5415 (1.5)	5722 (1.5)	5052 (1.4)	25,453 (1.5)	
Obstetric/gynaecological	2316 (0.9)	2316 (0.8)	2680 (0.7)	2956 (0.8)	2982 (0.8)	13,250 (0.8)	
Other health conditions ^†^	21,767 (8.3)	23,017 (8.0)	27,866 (7.7)	28,750 (7.5)	25,735 (7.1)	127,135 (7.7)	
Unclear problem	68,567 (26.2)	77,837 (27.0)	96,213 (26.6)	102,474 (26.8)	94,619 (26.1)	439,710 (26.5)	
Type of Rescue Vehicle Dispatched ^§^							
Basic EMV	178,346 (68.2)	195,047 (67.7)	246.663 (67.7)	266,550 (69.7)	247,751 (68.4)	1,134,357 (68.4)	<0.001
Advanced EMV type 1	72,892 (27.9)	80,614 (27.9)	101,652 (27.9)	102,399 (26.8)	97,305 (26.9)	454,862 (27.4)	
Advanced EMV type 2	9197 (3.5)	11,295 (3.9)	13,585 (3.7)	10,729 (2.8)	14,502 (4.0)	59,308 (3.6)	
Helicopter	1084 (0.4)	1332 (0.5)	2493 (0.7)	2664 (0.7)	2512 (0.7)	10,085 (0.6)	
Other	-	-	44 (0.0)	40 (0.0)	32 (0.0)	116 (0.0)	
Emergency Medical Services (EMS) Criticality Codes Assigned							
Red	18,829 (7.2)	21,121 (7.3)	26,248 (7.2)	25,067 (6.6)	24,154 (6.7)	115,419 (7)	<0.001
Yellow	77,710 (29.7)	88,885 (30.8)	112,591 (30.9)	117,337 (30.7)	116,328 (32.1)	512,851 (30.9)	
Green	156,474 (59.8)	169,327 (58.7)	216,415 (59.4)	229,311 (60.0)	211,235 (58.3)	982,762 (59.3)	
White	8506 (3.3)	8955 (3.1)	9183 (2.5)	10667 (2.8)	10,385 (2.9)	47,696 (2.9)	
Number of Rescue Vehicles Dispatched							
1	220,856 (84.4)	237,064 (82.2)	291.645 (80.0)	303,179 (79.3)	286,392 (79.1)	1,339,136 (80.7)	<0.001
2	38,900 (14.8)	48,602 (16.9)	67,158 (18.4)	72,960 (19.1)	69,760 (19.3)	297,380 (17.9)	
≥3	1763 (0.8)	2622 (0.9)	5634 (1.6)	6243 (1.6)	5950 (1.6)	22,212 (1.4)	
Number of patients transported per rescue vehicle							
None	61,300 (23.4)	68,404 (23.7)	92.465 (25.4)	99,269 (26.0)	96,873 (26.7)	418,311 (25.2)	<0.001
One person	197,440 (75.5)	216,918 (75.2)	267,869 (73.5)	279,054 (73.0)	261,477 (72.2)	1,222,868 (73.7)	
More than one person	2669 (1.0)	2966 (1.0)	4103 (1.1)	4059 (1.0)	3752 (1.0)	17,549 (1.1)	
Site of Rescue Completion							
Hospital ED	200,219 (76.6)	219,884 (76.3)	271,972 (74.6)	282,749 (73.9)	264,936 (73.2)	1,239,760 (74.7)	<0.001
Rendez vous	200 (0.1)	232 (0.1)	195 (0.1)	364 (0.1)	293 (0.1)	1284 (0.1)	
At home	99 (0.0)	22 (0.0)	22 (0.0)	35 (0.1)	33 (0.0)	211 (0.0)	
Morgue	12 (0.0)	13 (0.0)	26 (0.0)	19 (0.0)	17 (0.0)	87 (0.0)	
Other *	55,456 (21.2)	59,834 (20.7)	79,141 (21.7)	85,277 (22.3)	83,563 (23.0)	363,271 (21.9)	
Rescue cancelled	5533 (2.1)	8303 (2.9)	13,081 (3.6)	13,938 (3.6)	13,260 (3.7)	54,115 (3.3)	
Total Vehicle Dispatched	261,519	288,288	365,437	382,382	362,102	1,658,728	

EMV: emergency medical vehicle. EMS criticality codes—Red: need of an immediate response, life-threatening situation with a chance of survival; Yellow: rapid response, presence of a possibly life-threatening condition not in an immediate danger of death; Green: the situation is not an emergency, presence of injuries, acute but stable conditions; White: the situation is not an emergency, minor injuries not a life-threatening situation, medical care not often required. ^†^, Other health conditions: Tumours, Metabolic, Gastroenterological, Urological, Ophthalmological, Otolaryngological, Dermatological, Infectious. ^§^, Advanced EMVs carry one of two rescue teams: type (1) doctors and nurses with advanced life-saving capabilities, as well as specially trained volunteer rescuers or type (2) nurses with advanced life-saving capabilities as well as specially trained volunteer rescuers. Basic EMVs carry a rescue team of specially trained volunteer rescuers with basic life support capabilities. Rescue helicopter teams consist of an anaesthesiologist, a nurse, and a mountain rescue technician. *, Other: vehicle failure, patient not found, patient already transported, site unattainable.

**Table 3:** Adjusted Odds Ratios and 95% confidence interval (95%CI) of rescue completion in a hospital Emergency Department (ED) by type of rescue vehicle and Emergency Medical Services (EMS) criticality codes; Piedmont 2013–2017.

Criticality	Rescue Vehicle	OR	[95%CI]	OR
**Red**	EMV type1	1.00		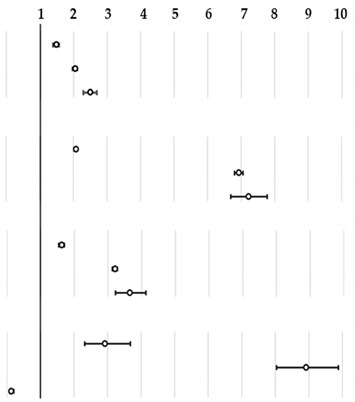
	EMV type2	1.47	[1.4–1.6]	
	Basic EMV	2.04	[2.0–2.1]	
	Helicopter	2.48	[2.3–2.7]	
**Yellow**	EMV type1	1.00		
	EMV type2	2.07	[2.0–2.1]	
	Basic EMV	6.93	[6.8–7.1]	
	Helicopter	7.22	[6.7–7.8]	
**Green**	EMV type1	1.00		
	EMV type2	1.62	[1.5–1.7]	
	Basic EMV	3.21	[3.1–3.3]	
	Helicopter	3.64	[3.2–4.1]	
**White**	EMV type1	1.00		
	EMV type2	2.92	[2.3–3.7]	
	Basic EMV	8.91	[8.0–9.9]	
	Helicopter	0.12	[0.1–0.2]	

## Supplementary Materials

The following are available online at https://www.mdpi.com/2227-9032/8/4/551/s1, Table S1: Proportions (within year percentage) and 95% Confidence Interval (95%CI) of Emergency Medical Services (EMS) calls for which at least one rescue vehicle was dispatched, by year (2013–2017): within year proportions and 95% Confidence Interval; Table S2: Characteristics of Emergency Medical Services (EMS) rescue vehicle dispatches by year (2013–2017) among 1,658,728 total rescue vehicles dispatched. Table S3: Emergency Medical Services (EMS) criticality codes* by type of rescue vehicle dispatched and rescue completion (RC) at a hospital emergency department (HED); percentage of rescues completed at HED. Total emergency medical vehicles (EMV) dispatched: 1,658,728, with 1,239,760 RC at HED; 2013–2017.
