# Supplementary material for: Trends and Characteristics of Emergency Medical Services in Italy: A 5-Years Population-Based Registry Analysis
Source: Healthcare (Basel). 2020 Dec 11;8(4):551. doi: 10.3390/healthcare8040551 (PMC7763006; doi:10.3390/healthcare8040551)
Supplement: Supplementary file 1 [file healthcare-08-00551-s001.pdf]

Table S1. Proportions (within year percentage) and 95% Confidence Interval (95%CI) of Emergency Medical Services (EMS) calls for which at least one rescue vehicle was dispatched, by year (2013–2017): within year proportions and 95% Confidence Interval.

Caller	2013	2014	2015	2016	2017	overall	2013–2017	*p*
	% (95% CI)	% (95% CI)	% (95% CI)	% (95% CI)	% (95% CI)	% (95% CI)	Difference %	Value
Private citizen	87.36 (87.23–87.49)	86.99 (86.86–87.12)	86.76 (86.64–86.88)	87.19 (87.07–87.30)	88.70 (88.59–88.81)	87.41 (87.36–87.47)	1.53	<0.001
Nursing home	3.40 (3.32–3.47)	3.41 (3.34–3.48)	3.48 (3.41–3.54)	3.04 (2.99–3.10)	3.10 (3.04–3.16)	3.27 (3.24–3.30)	–8.82	NS
Authority	2.89 (2.83–2.96)	3.10 (3.03–3.17)	3.22 (3.16–3.28)	3.54 (3.48–3.60)	2.62 (2.56–2.67)	3.09 (3.06–3.12)	–9.34	NS
GP/Paediatrician	2.51 (2.45–2.57)	2.60 (2.54–2.66)	2.44 (2.39–2.50)	2.23 (2.18–2.28)	1.73 (1.69–1.78)	2.28 (2.25–2.30)	–31.08	<0.05
EMS Physician	2.48 (2.42–2.54)	2.68 (2.62–2.74)	2.35 (2.30–2.40)	1.95 (1.90–1.99)	1.45 (1.41–1.49)	2.14 (2.12–2.17)	–41.53	<0.001
Private hospital	0.21 (0.20–0.23)	0.19 (0.18–0.21)	0.18 (0.16–0.19)	0.14 (0.13–0.15)	0.16 (0.15–0.17)	0.17 (0.17–0.18)	–23.81	NS
Other †	1.14 (1.10–1.19)	1.02 (0.98–1.06)	1.57 (1.53–1.61)	1.91 (1.87–1.96)	2.24 (2.19–2.30)	1.63 (1.61–1.65)	96.49	<0.001
**Call Time**								
7a.m.–6:59p.m.	64.64 (64.45–64.84)	64.92 (64.73–65.10)	65.43 (65.26–65.59)	65.17 (65.01–65.33)	65.34 (65.18–65.51)	65.13 (65.06–65.21)	1.08	<0.001
7p.m.–6:59a.m.	35.36 (35.16–35.55)	35.08 (34.90–35.27)	34.57 (34.41–34.74)	34.83 (34.67–34.99)	34.66 (34.49–34.82)	34.87 (34.79–34.94)	1.98	<0.05
**Call Location**								
Home	64.79 (64.60–64.98)	64.91 (64.73–65.10)	63.68 (63.52–63.85)	63.07 (62.91–63.23)	62.85 (62.68–63.02)	63.76 (63.68–63.83)	–2.99	<0.001
Street or highway	12.66 (12.53–12.80)	12.74 (12.61–12.87)	12.84 (12.73–12.96)	13.33 (13.22–13.45)	13.08 (12.96–13.19)	12.96 (12.90–13.01)	3.32	NS
Public building	3.50 (3.42–3.57)	3.48 (3.41–3.55)	3.65 (3.59–3.71)	3.71 (3.64–3.77)	3.64 (3.57–3.70)	3.60 (3.57–3.63)	4.00	NS
Industrial place	1.88 (1.83–1.94)	1.92 (1.87–1.98)	2.05 (2.00–2.10)	1.96 (1.91–2.00)	1.92 (1.87–1.97)	1.95 (1.93–1.97)	2.13	NS
Sport building	0.99 (0.95–1.03)	0.98 (0.95–1.02)	0.95 (0.92–0.98)	0.89 (0.86–0.92)	0.91 (0.88–0.95)	0.94 (0.93–0.96)	–8.08	NS
School	0.88 (0.84–0.91)	0.98 (0.94–1.02)	0.86 (0.83–0.89)	0.89 (0.85–0.92)	0.93 (0.90–0.96)	0.90 (0.89–0.92)	5.68	NS
Other locations *	15.31 (15.16–15.45)	14.99 (14.85–15.12)	15.97 (15.84–16.09)	16.16 (16.03–16.28)	16.68 (16.55–16.80)	15.89 (15.83–15.94)	8.95	<0.001
Total numbers	240,878	262,210	326,991	341,621	323,155	1,494,855	34.16	

† Other Callers comprise all calls originally labelled as "none of the above"; * Other Call locations comprise nursing homes, private hospitals, and residential care facilities; GP: general practitioner

Table S2. Characteristics of Emergency Medical Services (EMS) rescue vehicle dispatches by year (2013–2017) among 1 658 728 total rescue vehicles dispatched.

EMS dispatches	2013	2014	2015	2016	2017	overall	2013–2017	*p*
	% (95% CI)	% (95% CI)	% (95% CI)	% (95% CI)	% (95% CI)	% (95% CI)	Difference (%)	Value
Number of People Involved								
1	85.48 (85.35–85.62)	83.82 (83.68–83.95)	81.45 (81.33–81.58)	80.27 (80.14–80.40)	79.82 (79.69–79.95)	81.92 (81.86–81.98)	–6.62	<0.001
2	14.05 (13.92–14.18)	15.62 (15.48–15.75)	16.84 (16.72–16.96)	17.65 (17.53–17.77)	18.09 (17.96–18.22)	16.66 (16.60–16.71)	28.75	<0.001
>2	0.47 (0.44–0.49)	0.57 (0.54–0.60)	1.44 (1.40–1.48)	2.08 (2.04–2.13)	2.09 (2.04–2.14)	1.42 (1.41–1.44)	344.68	<0.001
Health Problem/Injury Reported by the Caller								
Traumatic	20.21 (20.06–20.37)	20.12 (19.97–20.26)	22.53 (22.39–22.66)	22.84 (22.71–22.97)	22.78 (22.64–22.91)	21.88 (21.82–21.95)	12.72	<0.001
Cardiocirculatory	15.48 (15.34–15.62)	15.06 (14.93–15.19)	14.55 (14.44–14.67)	14.49 (14.38–14.60)	16.13 (16.01–16.25)	15.13 (15.07–15.18)	4.2	<0.05
Neurological	12.27 (12.14–12.39)	12.53 (12.41–12.65)	11.97 (11.86–12.08)	11.93 (11.83–12.03)	11.27 (11.16–11.37)	11.96 (11.91–12.01)	–8.15	<0.001
Respiratory	11.67 (11.54–11.79)	11.58 (11.46–11.69)	11.44 (11.34–11.54)	11.00 (10.90–11.10)	11.28 (11.17–11.38)	11.37 (11.32–11.42)	–3.34	NS
Psychiatric	3.20 (3.13–3.27)	3.30 (3.23–3.36)	3.07 (3.01–3.13)	3.16 (3.10–3.21)	3.09 (3.04–3.15)	3.16 (3.13–3.18)	–3.44	NS
Unknown	2.46 (2.41–2.52)	2.26 (2.20–2.31)	1.92 (1.88–1.97)	2.14 (2.10–2.19)	2.38 (2.33–2.43)	2.22 (2.20–2.24)	–3.25	NS
Toxicological	1.74 (1.69–1.79)	1.63 (1.59–1.68)	1.48 (1.44–1.52)	1.50 (1.46–1.54)	1.40 (1.36–1.43)	1.53 (1.52–1.55)	–19.54	NS
Obstetric/gynaecological	0.89 (0.85–0.92)	0.80 (0.77–0.84)	0.73 (0.71–0.76)	0.77 (0.75–0.80)	0.82 (0.79–0.85)	0.80 (0.79–0.81)	–7.87	NS
Unclear problem	32.08 (31.90–32.26)	32.73 (32.55–32.90)	32.30 (32.15–32.45)	32.18 (32.03–32.33)	30.86 (30.71–31.01)	31.96 (31.88–32.03)	–3.8	<0.001
Type of Rescue Vehicle Dispatched §								
Basic EMV	68.20 (68.02–68.37)	67.66 (67.49–67.83)	67.50 (67.35–67.65)	69.71 (69.56–69.85)	68.42 (68.27–68.57)	68.39 (68.32–68.46)	0.32	NS
Advanced EMV type 1	27.87 (27.70–28.04)	27.96 (27.80–28.13)	27.82 (27.67–27.96)	26.78 (26.64–26.92)	26.87 (26.73–27.02)	27.42 (27.35–27.49)	–3.59	<0.001
Advanced EMV type 2	3.52 (3.45–3.59)	3.92 (3.85–3.99)	3.72 (3.66–3.78)	2.81 (2.75–2.86)	4.00 (3.94–4.07)	3.58 (3.55–3.60)	13.64	NS
Helicopter	0.41 (0.39–0.44)	0.46 (0.44–0.49)	0.68 (0.66–0.71)	0.70 (0.67–0.72)	0.69 (0.67–0.72)	0.61 (0.60–0.62)	68.29	<0.001
Other			0.01 (0.01–0.02)	0.01 (0.01–0.01)	0.01 (0.01–0.01)	0.01 (0.01–0.01)	–	
Number of Rescue Vehicles Dispatched								
1	84.45 (84.31–84.59)	82.23 (82.09–82.37)	79.81 (79.68–79.94)	79.29 (79.16–79.42)	79.09 (78.96–79.22)	80.73 (80.67–80.79)	–6.35	<0.001
2	14.87 (14.74–15.01)	16.86 (16.72–17.00)	18.38 (18.25–18.50)	19.08 (18.96–19.21)	19.27 (19.14–19.39)	17.93 (17.87–17.99)	29.59	<0.001
≥3	0.67 (0.64–0.71)	0.91 (0.88–0.94)	1.54 (1.50–1.58)	1.63 (1.59–1.67)	1.64 (1.60–1.69)	1.34 (1.32–1.36)	144.78	<0.05
Number of Patients Transported Per Rescue Vehicle								

None	23.44 (23.28–23.60)	23.73 (23.57–23.88)	25.30 (25.16–25.44)	25.96 (25.82–26.10)	26.75 (26.61–26.90)	25.22 (25.15–25.28)	14.12	<0.001
One person	75.50 (75.33–75.66)	75.24 (75.09–75.40)	73.30 (73.16–73.44)	72.98 (72.84–73.12)	72.21 (72.06–72.36)	73.72 (73.66–73.79)	–4.36	<0.001
More than one person	1.02 (0.98–1.06)	1.03 (0.99–1.07)	1.12 (1.09–1.16)	1.06 (1.03–1.09)	1.04 (1.00–1.07)	1.06 (1.04–1.07)	1.96	NS
Site of Rescue Completion								
Hospital	76.56 (76.40–76.72)	76.27 (76.12–76.43)	74.42 (74.28–74.57)	73.94 (73.80–74.08)	73.17 (73.02–73.31)	74.74 (74.68–74.81)	–4.43	<0.001
Rendez vous	0.08 (0.07–0.09)	0.08 (0.07–0.09)	0.05 (0.05–0.06)	0.10 (0.09–0.11)	0.08 (0.07–0.09)	0.08 (0.07–0.08)	0	NS
At home	0.04 (0.03–0.05)	0.01 (0.00–0.01)	0.01 (0.00–0.01)	0.01 (0.01–0.01)	0.01 (0.01–0.01)	0.01 (0.01–0.01)	-75	NS
Morgue	0.00 (0.00–0.01)	0.00 (0.00–0.01)	0.01 (0.00–0.01)	0.00 (0.00–0.01)	0.00 (0.00–0.01)	0.01 (0.00–0.01)	0	NS
Other *	21.21 (21.05–21.36)	20.75 (20.61–20.90)	21.66 (21.52–21.79)	22.30 (22.17–22.43)	23.08 (22.94–23.21)	21.90 (21.84–21.96)	8.82	<0.001
Rescue cancelled	2.12 (2.06–2.17)	2.88 (2.82–2.94)	3.58 (3.52–3.64)	3.65 (3.59–3.70)	3.66 (3.60–3.72)	3.26 (3.24–3.29)	72.64	<0.001
Total Vehicle Dispatched	261,519	288,288	365,437	382,382	362,102	1,658,728		

EMV: emergency medical vehicle; §Advanced EMVs carry one of two rescue teams: type 1) doctors and nurses with advanced life–saving capabilities, as well as specially trained volunteer rescuers or type 2) nurses with advanced life-saving capabilities as well as specially trained volunteer rescuers. Basic EMVs carry a rescue team of specially trained volunteer rescuers with basic life support capabilities. Rescue helicopter teams consist of an anaesthesiologist, a nurse, and a mountain rescue technician.

* Other: vehicle failure, patient not found, patient already transported, site unattainable.

Table S3. Emergency Medical Services (EMS) criticality codes* by type of rescue vehicle dispatched and rescue completion (RC) at a hospital emergency department (HED); percentage of rescues completed at HED. Total emergency medical vehicles (EMV) dispatched: 1 658 728, with 1 239 760 RC at HED; 2013–2017.

	EMS Criticality Codes				
**Type of Rescue Vehicle §**	**Red**	**Yellow**	**Green**	**White**	**Overall**
**RC at HED**	***N* (%)**	***N* (%)**	***N* (%)**	***N* (%)**	***N* (%)**
**RC at HED/Type of rescue vehicle**	%	%	%	%	%
Basic EMV	27 930 (2.5)	135 219 (11.9)	926 604 (81.7)	4 4604 (3.9)	1 134 357 (100)
RC at HED	13 992 (1.5)	102 031 (10.7)	799 761 (84.0)	36 421 (3.8)	952 205 (100)
RC at HED/all basic EMV	50.1	75.5	86.3	81.7	83.9
Doctor/nurse advanced EMV	78 146 (17.2)	328 392 (72.2)	45 956 (10.1)	2 368 (0.5)	454 862 (100)
RC at HED	34 718 (14.5)	182 468 (76.0)	22 269 (9.3)	665 (0.3)	240 120 (100)
RC at HED/all type 1 advanced EMV	44.4	55.6	48.5	28.1	52.8
Nurse advanced EMV	5 814 (9.8)	44 505 (75.0)	8 599 (14.5)	390 (0.7)	59 308 (100)
RC at HED	2 546 (6.3)	32 141 (79.3)	5 633 (13.9)	205 (0.5)	40 525 (100)
RC at HED/all type 2 advanced EMV	43.8	72.2	65.5	52.6	68.3
Helicopters	3 516 (34.9)	4 709 (46.7)	1 542 (15.3)	318 (3.1)	10 085 (100)
RC at HED	2 151 (31.1)	3 617 (52.4)	1 122 (16.2)	17 (0.2)	6 907 (100)
RC at HED/all Helicopters	61.2	76.8	72.8	5.3	68.5
Other vehicles	13 (11.2)	26 (22.4)	61 (52.6)	16 (13.8)	116 (100)
RC at HED	2 (66.7)	0 (0.0)	1 (33.3)	0 (0.0)	3 (100)
RC at HED/all other vehicles	15.4	0.0	1.6	0.0	2.6
Overall transports	115 419 (7.0)	512 851 (30.9)	982 762 (59.2)	47 696 (2.9)	1 658 728 (100)
Overall RC at HED	53 409 (4.3)	320 257 (25.8)	828 786 (66.8)	37 308 (3.0)	1 239 760 (100)
RC at HED/all overall transports	46.3	62.4	84.3	78.2	74.7

*, EMS criticality codes—Red: need of an immediate response, life–threatening situation with a chance of survival; Yellow: rapid response, presence of a possible life–threatening condition not in an immediate danger of death; Green: the situation is not an emergency, presence of injuries, acute but stable conditions; White: the situation is not an emergency, minor injuries not a life–threatening situation, medical care not often required. §, Type of recue vehicle: Advanced EMVs carry one of two rescue teams: type 1) doctors and nurses with advanced life–saving capabilities, as well as specially trained volunteer rescuers or type 2) nurses with advanced life–saving capabilities as well as specially trained volunteer rescuers. Basic EMVs carry a rescue team of specially trained volunteer rescuers with basic life support capabilities. Rescue helicopter teams consist of an anaesthesiologist, a nurse, and a mountain rescue technician. RC – rescue completion; HED – hospital emergency department.
